# Screening for orthorexia nervosa among nursing students: prevalence and associations between the Chinese version of the Düsseldorf Orthorexia Scale and the Chinese version of the Orthorexia Nervosa Inventory

**DOI:** 10.3389/fnut.2026.1767114

**Published:** 2026-03-23

**Authors:** Fan Yan, Yangyao Peng, Xinzhang Sun, Hanqing Zhang

**Affiliations:** 1 Department of Hematology, Henan Provincial People's Hospital, Zhengzhou, Henan, China; 2 Department of Cardiovascular Surgery, Zhongnan Hospital of Wuhan, Wuhan, Hubei, China; 3 Yangtze University, Health Science Center, Jingzhou, Hubei, China; 4 Faculty of Public Health, Mahidol University, Bangkok, Thailand

**Keywords:** eating disorder, nursing, orthorexia nervosa, prevalence, scale

## Abstract

**Aim:**

To estimate orthorexia nervosa (ON) screening prevalence and risk stratification among Chinese nursing students, examine associations of sex, grade and BMI with ON screening positivity, and describe symptom severity using the Chinese Orthorexia Nervosa Inventory (C-ONI).

**Design:**

Cross-sectional survey.

**Methods:**

Undergraduate nursing students (*n* = 693; effective response rate 96.25%) completed the Chinese Düsseldorf Orthorexia Scale (C-DOS) and C-ONI. ON screening positivity was defined as C-DOS ≥30 with three-level risk stratification. Wilson 95% CIs were calculated. Welch's ANOVA compared C-ONI scores across C-DOS strata. Multivariable logistic regression tested correlates (sex, grade, BMI). Agreement between dichotomized C-DOS and ONI (C-ONI ≥72) was examined.

**Results:**

C-DOS classified 76.0% as ON absent, 10.2% at risk, and 13.7% ON present (95% CI: 11.2%−16.5%). C-ONI total scores increased across strata (42.3 ± 10.2, 58.8 ± 8.7, 70.8 ± 11.9; all *p* < 0.001). Dichotomized agreement was 90.6% (628/693) with 9.4% discordance (7.9% C-DOS-only; 1.4% ONI-only). Females had lower odds of ON screening positivity than males (OR = 0.423, 95% CI: 0.264–0.678; *p* < 0.001); BMI and grade were not significant.

**Conclusions:**

ON screening positivity was 13.7% in Chinese nursing students with a clear risk–severity gradient. Although instruments largely agreed, non-overlap suggests prevalence estimates and individual classification depends on tool/cut-off; validation against clinical anchor criteria is needed.

## 1 Introduction

In recent years, increasing attention has been paid to health and healthy dietary habits across multiple scientific disciplines. Dietary behaviors not only influence physical growth and development but also have profound implications for overall health and wellbeing. A healthy diet is a prerequisite for good health, as it supports immune function and facilitates recovery. However, an excessive preoccupation with food quality may paradoxically become detrimental to health. The term orthorexia nervosa (ON) was first introduced in 1997 by Bratman (1) to describe a pattern of behavior characterized by an obsessive pursuit of dietary purity. This excessive preoccupation with the quality of food may ultimately lead to the development of a distinct form of eating disorder. The term is derived from the Greek words orthós, meaning “correct” or “right,” and orexis, meaning “appetite.” Orthorexia Nervosa (ON) has been conceptualized as a potential emerging disorder, and based on current understandings, diagnostic criteria have been proposed (2, 3). As summarized and discussed by Cena et al. (4) and Dunn and Bratman (5), three primary indicators have been emphasized. Broadly, these include: (1) compulsive behaviors and obsessive thoughts concerning ostensibly healthful dietary practices; (2) extreme emotional distress resulting from violations of self-imposed restrictive dietary rules; and (3) physical and psychosocial impairment (e.g., malnutrition, and compromised social, academic, or occupational functioning). With respect to dietary restriction, orthorexia nervosa (ON) shares similarities with two eating disorders—anorexia nervosa (AN) and avoidant/restrictive food intake disorder (ARFID)—although their underlying motivations differ. Individuals with AN primarily restrict overall food intake and high-calorie foods in order to achieve or maintain a markedly low body weight, even though such restrictive behaviors may be framed as the pursuit of healthy eating to enhance social acceptability. In contrast, individuals with ARFID restrict food intake mainly on the basis of sensory characteristics of food, previous aversive eating experiences, or a lack of interest in eating (6). By comparison, ON is thought to be driven by concerns about the perceived quality of food (e.g., its healthfulness or purity), rather than by weight- or shape-related concerns (7). Orthorexia nervosa describes an extreme pattern of eating adopted in the pursuit of health, characterized primarily by obsessive thoughts about “healthy foods” and compulsive eating-related behaviors (8). At present, ON has no widely accepted official definition and is not listed in official ICD-11 (9) or DSM-V (10). Though, there're amount of diagnostic criteria for ON have been proposed (11), most of them are criticized by researchers or have not been verified. As a result, there's no standardized diagnostic criteria and treatment regime (12).

The incidence of ON varies by country and population, and data on ON prevalence depend on the validity and reliability of the tools used for assessment (13). Research has previously identified that students within the health science field demonstrate an increased prevalence of disordered eating (14).

Previous studies involving nurses and nursing students have shown that, among nursing students, stronger orthorexia tendencies are associated with a greater likelihood of using dietary supplements, many of which are not taken based on recommendations from healthcare professionals, suggesting potential health risks and insufficient nutrition-related knowledge. Among clinical nurses, orthorexia tendencies have been found to be associated with higher levels of cyberchondria, such that greater cyberchondria is linked to stronger orthorexia tendencies, possibly reflecting an intensified pursuit of “correct,” planned, and cautious eating behaviors (15, 16). Orthorexia tendency has been reported in nursing-related populations, although estimates vary considerably due to differences in instruments and cut-off criteria. In a sample of candidate doctors and candidate nurses, Yilmazel (17) assessed orthorexia tendency using the ORTO-15 and applied a cut-off of <40; overall, 62.2% of participants were classified as having orthorexia tendency. In that study, nursing students constituted 58.3% of the sample, and subgroup comparisons showed an orthorexia tendency rate of 54.5% among candidate nurses (vs. 73.0% among candidate doctors). Among nursing students specifically, Aktürk et al. (18). investigated 558 nursing students using the ORTO-15 and classified orthorexia tendency based on a Turkish validation cut-off of ≤ 33. They reported that 73.5% of nursing students met the convergent validity for orthorexia tendency, with a mean ORTO-15 score of approximately 36.8 ± 1.2.

Previous studies of Orthorexia Nervosa in China have mostly focused on scale validation (19–21), and the sample sizes are largely from university students (22). However, no surveys have been conducted among healthcare professionals such as nutrition and nursing. A previous study using the Chinese version of the 10-item DOS (C-DOS) found that the prevalence of ON was 7.8% (and reported a higher prevalence in men than women: 10.6 vs. 5.3%). Specific data (*n* = 1,075): 84 people were ON present; 196 were ON at risk; and 795 were ON absent (20). Another study used the ORTO-15 scale with a cutoff of 40, and the results for the college student sample were: “prevalence of ON assessed by ORTO-15 was 46.7%” (21). Although research on orthorexia nervosa (ON) among university students has been increasing, empirical evidence regarding the screening prevalence, risk stratification, and associated factors of ON in Chinese nursing students remains limited, particularly studies that jointly apply the Düsseldorf Orthorexia Scale (C-DOS) (20) and the Chinese version of Orthorexia Nervosa Inventory (C-ONI) (19) to examine the consistency of risk stratification across instruments. Therefore, this study aimed to jointly use the C-DOS and ONI in a sample of Chinese nursing students to systematically describe the screening prevalence and risk stratification of ON; to further compare differences and gradient patterns in ONI scores across C-DOS-defined risk levels in order to verify the consistency of risk stratification between the two instruments; and to explore the associations of sex, academic grade, and body mass index (BMI) with C-DOS–defined ON present status using multivariable analyses. The findings are expected to provide empirical evidence to support early identification, risk assessment, and the development of targeted health education and intervention strategies for Chinese nursing students.

## 2 Method

This study employed a cross-sectional survey design to assess the distribution characteristics of eating disorder-related manifestations among nursing students and to explore their association with demographic factors and physical indicators.

### 2.1 Sample size

This cross-sectional study aimed to estimate the screening-positive rate of orthorexia nervosa (ON) among nursing students and to examine associated factors; therefore, the sample size was justified to satisfy both prevalence precision and multivariable model stability requirements. For prevalence estimation, we used the single-proportion formula (*n* = *Z*^{2}^*p*(1–*p*)/*d*^{2}^) with a two-sided 95% confidence level (*Z* = 1.96) and an absolute precision of (*d* = 0.04) (23). To address uncertainty in the expected screening-positive rate, calculations were performed using (*p* = 0.078) based on prior student studies and an upper-bound scenario (*p* = 0.50; maximizing [*p*(1–*p*)] and thus the required sample size), and the larger requirement was adopted; the target sample size was then inflated by 10% to account for incomplete or invalid questionnaires (24). Therefore, the minimum required sample sizes are approximately 173 and 600, respectively, so 600 is adopted as the minimum effective sample size; considering approximately 10% invalid questionnaires, the planned sample size is increased to approximately 667. For multivariable binary logistic regression, we evaluated feasibility using the events-per-variable (EPV) principle (rule of thumb EPV ≥10) derived from simulation studies and widely cited guidance; given that grade would be dummy-coded, the planned model was expected to involve approximately six parameters. Because sparse categories can lead to unstable estimates, we prespecified that grade levels with very small cell counts would be collapsed (or model degrees of freedom reduced) if required to improve estimation stability (25).

### 2.2 Sampling procedure

Data collection was conducted between March and May 2025. With the permission of the university administration and relevant department heads, the research team approached eligible nursing students across all academic years (years 1–5) during scheduled classroom sessions or department assemblies. The principal investigator or a trained research assistant presented the study objectives, voluntary nature of participation, anonymity procedures, and informed consent details to potential participants. All students present in the selected sessions were invited to participate. The inclusion criteria were: (1) officially enrolled as an undergraduate nursing student; (2) able to comprehend and complete the Chinese questionnaire independently; and (3) provision of informed consent. Exclusion criteria included: (1) self-reported history of diagnosed eating disorders or severe mental illness; (2) current use of psychotropic medications that could significantly affect appetite or eating behavior.

### 2.3 Data collection and quality control

Eligible and consenting participants completed a self-administered, paper-based questionnaire packet in a supervised setting, which took approximately 15–20 min. To ensure data quality, investigators were present to answer procedural questions without influencing responses. Completed questionnaires were checked on-site for major omissions. All data were subsequently double entered into a secure electronic database by two independent research assistants, with discrepancies resolved by referring to the original forms.

### 2.4 Collected data and study instruments

(1) Demographic and anthropometric characteristics

Participants reported basic demographic information including sex and grade. Height and weight were self-reported and used to calculate body mass index (BMI). BMI was computed as: BMI = weight (kg)/height (m)^2^. When height was recorded in centimeters, it was first converted to meters before calculating BMI.

(2) Düsseldorf Orthorexia Scale (Chinese version, C-DOS) (20)

The Chinese version of the Düsseldorf Orthorexia Scale (C-DOS) was used to assess orthorexia-related tendencies. The C-DOS showed good internal consistency with an ordinal alpha of 0.80, and it also had good test-retest reliability of 0.77. The scale consists of 10 items, each rated on a four-point Likert scale (1 = strongly disagree, 4 = strongly agree). The total score is obtained by summing all item scores and ranges from 10 to 40, with higher scores indicating stronger orthorexia tendencies. In line with recommended cut-offs, participants were categorized into three groups based on their C-DOS scores: absence, at-risk, and ON present. In the present study, these C-DOS–based groups were used to describe the distribution of orthorexia-related status and served as the primary categorical variable for subsequent group comparisons and regression analyses. ON present was defined as a C-DOS total score ≥30.

(3) Orthorexia Nervosa Inventory (Chinese version, C-ONI) (19)

The Chinese version of the Orthorexia Nervosa Inventory (C-ONI) was used to assess the severity of orthorexia nervosa symptoms. The scale comprises 24 items, each rated on a four-point Likert scale (1 = strongly disagree, 4 = strongly agree). The total score is calculated as the sum of all item scores and ranges from 24 to 96, with higher scores reflecting more severe orthorexia-related symptomatology. In this study, the C-ONI total score was treated as a continuous variable and used to examine differences and trends across the three C-DOS groups.

### 2.5 Statistical analysis

All statistical analyses were conducted using Jamovi 2.3.28 (The Jamovi Project, Sydney, NSW, Australia) software, with a two-sided significance level set at α = 0.05. Continuous variables are presented as mean ± standard deviation (Mean ± SD) or median (interquartile range), and categorical variables as frequency and percentage [*n* (%)]. Wilson score intervals were used for 95% CIs around proportions. First, descriptive statistics were computed to summarize the sample and estimate the prevalence across C-DOS categories (absent ON, At Risk, ON Present). To compare C-ONI total scores across these categories, Welch's one-way ANOVA followed by Games-Howell *post-hoc* tests was employed due to unequal group sizes and potential heteroscedasticity, with variance homogeneity assessed by Levene's test. To identify factors associated with ON, a multivariate binary logistic regression model was fitted with ON status as the dependent variable and gender, academic year, and BMI as independent variables. Results are reported as odds ratios (ORs) with 95% CIs. Model fit was evaluated using the likelihood ratio chi-square test and McFadden's pseudo-*R*^2^, and multicollinearity was assessed by examining Variance Inflation Factor (VIF) values. Agreement between dichotomous classifications of the Chinese Düsseldorf Orthorexia Scale (C-DOS; ≥30 indicating presence) and the Chinese Orthorexia Nervosa Inventory (C-ONI; ≥72 indicating presence) was evaluated using Cohen's kappa coefficient. In addition to kappa, the raw agreement rate was calculated to describe the proportion of identical classifications. Cohen's kappa was interpreted according to conventional benchmarks, with values of 0.41–0.60 indicating moderate agreement.

## 3 Results

### 3.1 Sample characteristics

This study distributed 720 questionnaires and collected 693 valid responses, yielding an effective response rate of 96.25%. The majority of the included nursing students were female and in their second academic year. Key descriptive statistics for body mass index (BMI) and scores on the eating behavior assessment scales are summarized in Table 1.

**Table 1:** Characteristics of the study sample (*N* = 693).

**Variable**	**Statistics**
**Gender**, ***n*** **(%)**	
Female	547 (78.9%)
Male	146 (21.1%)
**Academic year**, ***n*** **(%)**	
Year 1	93 (13.4%)
Year 2	366 (52.8%)
Year 3	176 (25.4%)
Year 4 and above	58 (8.4%)
CDOS total score	20.5 ± 6.75 [20.0]
Body mass index (BMI, kg/m^2^)	21.8 ± 5.11 [20.6]
Total ONI score	47.9 ± 14.6 [47.0]

### 3.2 The prevalence of ON

Based on the C-DOS cut-offs, 76.0% of students were classified as absence (95% CI: 72.7%−79.2%), 10.2% as at risk (95% CI: 8.1%−12.7%), and 13.7% as ON present (95% CI: 11.2%−16.5%). The specific data are shown in Table 2.

**Table 2:** Frequencies of PRESENT(C-DOS).

**Present**	**Counts**	**% of total**	**Cumulative %**
1 absent	527	76.0%	76.0%
2 at-risk	71	10.2%	86.3%
3 present	95	13.7%	100.0%

### 3.3 Differences in ONI scores across C-DOS groups

Pearson's correlation showed a strong positive association between C-ONI and C-DOS [*r* = 0.869, 95% CI (0.849, 0.886), *p* < 0. 001]. Welch's one-way analysis of variance revealed a significant difference in C-ONI total scores across the three C-DOS groups [*F*_(2, 138)_ = 307.0, *p* < 0.001). Mean C-ONI scores increased progressively from the absence group (42.3 ± 10.2) to the at-risk group (58.8 ± 8.7) and the ON present group (70.8 ± 11.9). Games–Howell *post hoc* tests showed that all pairwise comparisons were statistically significant (all *p* < 0.001). The specific data are shown in Tables 3, 4.

**Table 3:** Results of correlation analysis between C-ONI and C-DOS total scores.

**Correlation matrix**			
		**CDOS_total**	**Total ONI**
CDOS_total	Pearson's *r*	—	
	df	—	
	*p*-Value	—	
	95% CI upper	—	
	95% CI lower	—	
	*N*	—	
Total ONI	Pearson's *r*	0.869^***^	—
	df	691	—
	*p*-Value	<0.001	—
	95% CI upper	0.886	—
	95% CI lower	0.849	—
	*N*	693	—

^***^p < 0.001.

**Table 4:** Differences and *post hoc* comparisons of C-ONI total scores across three C-DOS categories.

**One-way ANOVA**				
**One-way ANOVA (Welch's)**				
	***F***	**df1**	**df2**	***P*** **-value**
Total ONI	307	2	138	<0.001
***post hoc* tests**
Games-Howell *post-hoc* test—total ONI
		1	2	3
1	Mean difference	—	−16.5	−28.5
	*P*-value	—	<0.001	<0.001
2	Mean difference		—	−12.0
	*P*-value		—	<0.001
3	Mean difference			—
	*P*-value			—

High-risk status according to the Orthorexia Nervosa Inventory (ONI) was defined as a total score ≥72 and was compared with ON presence defined by a Chinese Düsseldorf Orthorexia Scale (C-DOS) total score ≥30. Concordance between the primary ON screening tool (C-DOS) and the alternative instrument (ONI) was examined based on dichotomous classifications. A strong and statistically significant association was observed between the two screening outcomes [χ^2^_(1)_ = 200, *p* < 0.001]. Participants identified as high risk by the ONI were substantially more likely to be screened positive by the C-DOS [odds ratio = 42.8, 95% CI (20.3, 90.2)]. Overall, the two instruments yielded concordant classifications (both positive or both negative) in 90.6% (628/693) of cases. Discordant classifications were observed in 65 participants, with 55 individuals (7.9% of the total sample) screened positive only by the C-DOS and 10 individuals (1.4%) screened positive only by the ONI. When agreement beyond chance was considered, Cohen's kappa indicated moderate agreement between the two instruments (κ = 0.50), suggesting that a substantial proportion of the observed concordance was attributable to chance rather than true agreement. The specific data are shown in Table 5.

**Table 5:** Agreement between C-DOS and ONI high-risk screening classifications.

**Contingency tables**			
	**PRESENT(C-DOS)**		
**PRESENT(C-ONI)**	**0**	**1**	**Total**
0	588	55	643
1	10	40	50
Total	598	95	693
χ^2^ **tests**			
	**Value**	**df**	***P*** **-value**
χ^2^	200	1	<0.001
*N*	693		
**Comparative measures**			
	**95% confidence intervals**		
	**Value**	**Lower**	**Upper**
Odds ratio	42.8	20.3	90.2
**Nominal**
	**Value**
Phi-coefficient	0.537
Cramer's *V*	0.537

### 3.4 Logistic regression analysis of factors associated with ON presence

A binomial logistic regression was performed to identify factors associated with screening positive for orthorexia nervosa (ON), defined by a C-DOS score ≥ 30. The model was statistically significant (χ^2^ = 15.6, df = 6, *p* = 0.016), indicating that the predictors collectively differentiated between the ON-present and ON-absent groups, although the explained variance was low (McFadden's *R*^2^ = 0.028). No multicollinearity was detected (all VIFs <1.01).

Gender was the only significant predictor. Male nursing students had 2.36 times higher odds of screening positive for ON compared to their female counterparts [OR = 2.36, 95% CI (1.47, 3.78), *p* < 0.001]. Neither BMI [OR = 1.02, 95% CI (0.98, 1.06), *p* = 0.351] nor year of study (all ps > 0.05, with year 1 as reference) showed a significant association with ON status. The specific data are shown in Tables 3, 6.

**Table 6:** Binary logistic regression coefficients for predicting C-DOS positive screening outcome.

**Binomial logistic regression**				
**Model fit measures**				
**Model**	**Deviance**	**AIC**	**BIC**	***R*** ^2^_McF_
1	540	552	579	0.0255
**Model coefficients—PRESENT(C-DOS)**							
						**95% confidence interval**	
**Predictor**	**Estimate**	**SE**	***Z***	***P*** **-value**	**Odds ratio**	**Lower**	**Upper**
Intercept	−2.53131	0.5677	−4.45854	<0.001	0.0796	0.0261	0.242
BMI	0.01954	0.0210	0.93187	0.351	1.0197	0.9787	1.063
**Grade**	
2–1	−0.00189	0.3428	−0.00551	0.996	0.9981	0.5097	1.954
3–1	0.03723	0.3736	0.09965	0.921	1.0379	0.4990	2.159
4–1	0.25658	0.4650	0.55180	0.581	1.2925	0.5196	3.215
**Sex**	
1–2	0.85935	0.2404	3.57393	<0.001	2.3616	1.4741	3.783

Estimates represent the log odds of “PRESENT(C-DOS) = 1” vs. “PRESENT(C-DOS) = 0.” Sex: male = 1 vs. female = 2.

## 4 Discussion

In this sample, the prevalence of orthorexia nervosa (ON) identified using the C-DOS was 13.7%, indicating that ON-related risk is not uncommon among the study population. The significant increase in C-ONI scores across ascending C-DOS risk categories provides empirical support for the convergent validity of the C-DOS, suggesting that higher risk classifications on the C-DOS are consistently associated with more pronounced orthorexia features as measured by an independent instrument. Although the C-DOS and C-ONI were highly correlated, their incomplete agreement in identifying individuals at high risk for ON suggests that the two instruments may capture overlapping but not identical aspects of orthorexia tendencies, underscoring the importance of cautious interpretation when using different screening tools.

(1) This study found a 13.7% positive rate for ON screening among nursing students, which is lower than the co-occurrence rate (27.5%) (26) reported in a recent meta-analysis in the general population. However, this review is primarily based on the ORTO-15 questionnaire, and numerous studies have indicated that ORTO-15 may overestimate the prevalence due to psychometric issues (27, 28). Meanwhile, based on the previous positive rate of 7.8% (20) obtained using DOS, and the positive rate of 13.7% in the nursing population, this also confirms that the prevalence of ON is significantly higher among people who study or work in the health field than in the general population (14) (This may be related to differences in sample structure, time, and environment). This phenomenon can be attributed to multiple factors: The self-selection mechanism may make individuals who already have a high level of concern for health and diet more inclined to choose the nursing profession (29). The study by Yilmazel reported an orthorexia tendency rate of 54.5% among candidate nurses in Turkey, while research by Aktürk et al. found an even higher prevalence of 73.5% within a similar nursing student population. This substantial discrepancy in findings compared to the present study is primarily attributed to the variation in measurement instruments, specifically the reliance on the ORTO-15 questionnaire. A growing body of research has challenged the validity of the ORTO-15, suggesting that it may fail to reflect the true prevalence of orthorexia nervosa (ON) and frequently leads to overestimation due to inherent psychometric limitations (30, 31).

In addition, male sex emerged as a significant risk factor for ON with nursing student, which is in line with previous research highlighting gender-related differences in health-oriented eating behaviors (20). In contrast to findings from other countries—where female nursing students have been reported to show higher prevalence rates of orthorexia nervosa (18)—our study revealed an opposite pattern, with male students demonstrating a significantly higher risk (OR = 2.36, 95% CI: 1.47–3.78).

(2) As a psychological and behavioral disorder, ON is more likely to be driven by psychological factors rather than physiological factors (weight), which is consistent with the findings of many studies (32–35).
(3) Consistency between C-DOS and C-ONI

The two tools demonstrated a highly significant statistical association in binary classification [χ^2^_(1)_ = 200, *p* < 0.001; OR = 42.8, 95% CI: 20.3–90.2]; however, their agreement was only moderate [κ = 0.50], with 65 cases (9.4%) being classified discordantly. This pattern indicates that, despite the significant association, the two tools are not interchangeable for classifying individuals. Importantly, the observed asymmetry in discordant classifications—where C-DOS identified significantly more positive cases than ONI—should not be interpreted as one tool being superior to the other. Rather, in the absence of a diagnostic gold standard, this discrepancy reflects fundamental differences between the tools in their theoretical focus, item content, and cutoff criteria. Consequently, different screening tools may yield markedly different epidemiological profiles of orthorexia nervosa risk within the same population (13, 27). This finding empirically underscores a core limitation highlighted in recent systematic reviews: research in this field is constrained by the use of heterogeneous assessment tools with varied psychometric foundations. Therefore, the 13.7% prevalence rate reported in this study should be explicitly interpreted as an estimate based on the C-DOS tool, and not as a definitive indicator of clinically significant anorexia nervosa.

Critical progress in future research will not be achieved merely through further cross-tool comparisons, but rather through the development of dysfunction-based, clinically anchored diagnostic criteria. Validating screening tools against such criteria is essential to establish true convergent validity and, ultimately, to define a diagnostic gold standard.

## 5 Implications for nursing practice

As healthcare professionals, nurses play an important role in promoting healthy eating habits in society. Assessing the status of Orthorexia Nervosa (ON) among nursing students is crucial to prevent self-prescription of related adverse health outcomes. Adolescence is a critical period for establishing healthy lifestyle habits. Identifying ON tendencies among nursing students allows for targeted interventions to guide them toward developing a balanced view of nutrition. It is recommended that nursing education programs incorporate relevant coursework emphasizing to educate students on proper usage, safe dosage limits, and potential side effects. At the same time, regular screening for ON tendencies should be implemented. Measures should be designed to reduce the social and psychological complications that may arise in high-risk individuals, with referrals to specialized institutions for clinical diagnosis and treatment when necessary.

Furthermore, seminars and thematic lectures can be organized to enhance students' understanding of ON. In nutrition education, attention should be given to alleviating excessive dietary anxiety and providing corresponding psychological support. Given that orthorexia nervosa is a relatively new concept, further exploration of effective intervention methods is warranted. Scientific assessment of students' dietary attitudes, health status, and the influence of family eating environments should be conducted, combined with clinical and psychological evaluations to provide comprehensive support.

## 6 Limitations

This study employed a cross-sectional design, making it impossible to infer causal relationships between variables. For example, although a male gender was found to be associated with a higher risk of ON, it was unclear whether this was a direct effect of gender itself or due to gender-related sociocultural, psychological, or environmental factors. While the sample size met the pre-specified target, internal imbalances existed: the high percentage of females (78.9%) and uneven grade distribution (52.8% were second-year students, with only four fifth-year students) limited the generalizability of the findings among male nursing students and students at different academic levels. The findings of the present study are highly dependent on the choice of measurement instrument and its associated cut-off value. We employed the Chinese version of the Düsseldorf Orthorexia Scale (C-DOS) as our primary assessment tool, using a cut-off score of ≥30 to identify orthorexia tendencies. However, the field of orthorexia nervosa currently lacks a universally accepted diagnostic gold standard, and different instruments [e.g., ORTO-15, EHQ (36), ONI] are grounded in distinct theoretical constructs and item formulations, which may yield substantially different prevalence estimates. In the current study, the C-DOS and the ONI produced discordant classifications in 65 participants (9.4%), with a markedly asymmetric distribution of results. This discrepancy itself underscores the potential classification bias inherent in reliance on a single self- report instrument.

## 7 Conclusion

This study, based on a survey of 693 nursing students, preliminarily delineates the prevalence and associated factors of Orthorexia Nervosa (ON) within this population. Screening using the C-DOS with a cut-off score of ≥30 revealed a positive screening rate for ON of 13.7%. Numerically, this proportion is higher than the 7.8% reported in previous validation studies targeting the general Chinese university student population using the same tool and cut-off. Therefore, under the premise of employing the same instrument and identical cut-off value, our findings suggest that the detection level of ON among nursing students may be higher than that in the general university student population. This warrants further validation of its robustness and generalizability in larger-scale, multi-center studies.

Multivariate analysis identified male gender as a significant independent risk factor for ON, with the risk being approximately 2.36 times higher than that for female. Furthermore, A significant difference in C-DOS scores was found across the three categories: “ON absent,” “ON at risk,” and “ON present.” supporting the scale's discriminant validity. However, a comparison with another commonly used instrument, the ONI, revealed that while the two were highly correlated, there was a 9.4% diagnostic inconsistency rate, characterized by a markedly asymmetric distribution. This contradictory outcome is not attributable to random error but rather serves as direct evidence exposing the inherent instability and limitations of relying on a single self-report tool for defining ON in the absence of a diagnostic gold standard.

## Data Availability

The original contributions presented in the study are included in the article/supplementary material, further inquiries can be directed to the corresponding author.
